# Supplementary figures and images for: The Effect of Exercise Training on Myocardial and Skeletal Muscle Metabolism by MR Spectroscopy in Rats with Heart Failure
Source: Metabolites. 2019 Mar 19;9(3):53. doi: 10.3390/metabo9030053 (PMC6468534; doi:10.3390/metabo9030053)

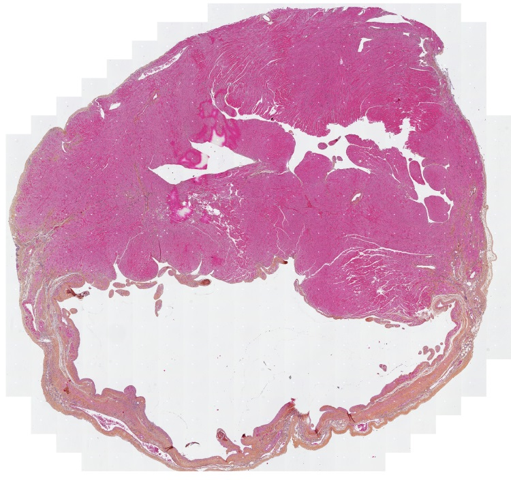

Supplement: Supplementary file 1 [file metabolites-09-00053-s001.zip › Figure S1.tif]
